# Reported Direct and Indirect Contact with Dromedary Camels among Laboratory-Confirmed MERS-CoV Cases

**DOI:** 10.3390/v10080425

**Published:** 2018-08-13

**Authors:** Romy Conzade, Rebecca Grant, Mamunur Rahman Malik, Amgad Elkholy, Mohamed Elhakim, Dalia Samhouri, Peter K. Ben Embarek, Maria D. Van Kerkhove

**Affiliations:** 1 Department of Infectious Hazard Management, Health Emergencies Programme, World Health Organization, 1211 Geneva, Switzerland; romy.conzade@helmholtz-muenchen.de (R.C.); grantr@who.int (R.G.); 2 Helmholtz Zentrum München, German Research Center for Environmental Health (GmbH), Institute of Epidemiology, 85764 Neuherberg, Germany; 3 Institut Pasteur, Centre for Global Health Research and Education, 75015 Paris, France; 4 Department of Infectious Hazard Management, Health Emergencies Programme, World Health Organization Regional Office for the Eastern Mediterranean, 11371 Cairo, Egypt; malikm@who.int (M.R.M.); elkholya@who.int (A.E.); elhakimm@who.int (M.E.); 5 Department of Country Preparedness and International Health Regulations, World Health Organization Regional Office for the Eastern Mediterranean, 11371 Cairo, Egypt; samhourid@who.int; 6 Department of Food Safety and Zoonoses, World Health Organization, 1211 Geneva, Switzerland; benembarekp@who.int

**Keywords:** MERS-CoV, dromedary camels, zoonotic transmission

## Abstract

Dromedary camels (*Camelus dromedarius*) are now known to be the vertebrate animal reservoir that intermittently transmits the Middle East respiratory syndrome coronavirus (MERS-CoV) to humans. Yet, details as to the specific mechanism(s) of zoonotic transmission from dromedaries to humans remain unclear. The aim of this study was to describe direct and indirect contact with dromedaries among all cases, and then separately for primary, non-primary, and unclassified cases of laboratory-confirmed MERS-CoV reported to the World Health Organization (WHO) between 1 January 2015 and 13 April 2018. We present any reported dromedary contact: direct, indirect, and type of indirect contact. Of all 1125 laboratory-confirmed MERS-CoV cases reported to WHO during the time period, there were 348 (30.9%) primary cases, 455 (40.4%) non-primary cases, and 322 (28.6%) unclassified cases. Among primary cases, 191 (54.9%) reported contact with dromedaries: 164 (47.1%) reported direct contact, 155 (44.5%) reported indirect contact. Five (1.1%) non-primary cases also reported contact with dromedaries. Overall, unpasteurized milk was the most frequent type of dromedary product consumed. Among cases for whom exposure was systematically collected and reported to WHO, contact with dromedaries or dromedary products has played an important role in zoonotic transmission.

## 1. Introduction

Middle East respiratory syndrome coronavirus (MERS-CoV) was first detected in a patient living in the Kingdom of Saudi Arabia (KSA) in September of 2012 [1]. Subsequent cases have included human infections across the Arabian Peninsula, occasional importation of cases outside the Arabian Peninsula, and associated clusters in other regions of the world. Outbreaks of non-sustained, human-to-human transmission have primarily occurred in healthcare settings [2].

MERS-CoV appears to be transmitted inefficiently between humans through casual contact in the general community [3]. In contrast, limited human-to-human transmission has occurred in healthcare settings from unprotected contact with or inadequate infection prevention and control measures in the care of MERS patients [4,5,6,7,8,9,10]. Health-care associated outbreaks have, on occasion, resulted in large outbreaks with significant public health and economic consequences, and health-care associated infections account for approximately half of the MERS-CoV infections reported to WHO to date. There have been significant improvements in implementation of adequate and specific infection prevention and control measures during healthcare outbreaks and these efforts have been shown to reduce human-to-human transmission in healthcare settings [11]. Despite this, substantial work remains to understand and prevent MERS-CoV from entering the human population and thus reducing the potential for amplified transmission in healthcare settings.

There is now strong consensus that dromedary camels (*Camelus dromedarius*) are the main source of transmission of MERS-CoV to humans. Despite MERS-CoV causing little to no disease in dromedaries, MERS-CoV can circulate within and between dromedary herds, and from dromedaries to humans [12]. MERS-CoV RNA has been detected, and viable virus has been isolated from dromedaries, including partial MERS-CoV genome sequences [13,14,15,16,17,18,19,20]. Analysis of outbreaks associated with a barn in KSA [21,22,23] and in Qatar [17,24] inferred animal-to-human transmission following parallel dromedary and human infections with nearly identical strains of MERS-CoV. Field surveys have been conducted on several domestic and wildlife species, including dromedary and Bactrian camels (*Camelus bactrianus*), goats, bats, cattle, sheep, chickens, swine, ducks, buffalo, and equids. Among dromedaries, seroprevalence field surveys have been conducted in a number of countries, including Australia [25], Burkina Faso [26], Egypt [27], Ethiopia [26], Jordan [28,29], Kazakhstan [30], Kenya [31], KSA [14,21,32], Mali [33], Morocco [26], Netherlands, Nigeria [34], Oman [35], Pakistan [36], Qatar [17], Somalia [37], Spain (Canary Islands) [34], Sudan [37], Tunisia [34], and the United Arab Emirates [38]. To date, MERS-CoV RNA or MERS-CoV-specific antibodies have been identified in dromedaries in all the countries mentioned above, except Australia, Kazakhstan, and the Netherlands.

Details as to the specific mechanism(s) of zoonotic transmission from dromedaries to humans remain unclear. Routes of camel-to-human MERS-CoV transmission likely include direct exposure to infected dromedaries and/or from an indirect exposure, including droplets of respiratory secretions from infected animals, or possibly through consumption of unprocessed and contaminated animal products, such as urine, unpasteurized milk, or raw meat [39]. Detailed epidemiological data on non-human exposures, such as direct or indirect animal exposure, have thus far been limited to a few case-control studies [40]. Studying MERS-CoV at the animal-human interface, and the routes of zoonotic transmission, highlights the need for a One Health approach to this research [41].

The International Health Regulations (2005) require all laboratory-confirmed MERS-CoV infections to be reported to the World Health Organization (WHO), and between 2012 and 30 June 2018, 2229 laboratory confirmed MERS-CoV infections have been reported to WHO. Since 2015, there have been notable improvements in surveillance and reporting of human cases, and since 2015, individual case data reported to WHO systematically includes information on travel history, contact with a confirmed or probable human MERS case, contact with dromedaries, and visits to human healthcare facilities within the 14 days prior to symptom onset. The aim of this study was to use epidemiological data of all MERS-CoV infections reported to WHO from 1 January 2015 to describe direct and indirect contact with dromedaries among all cases, and then separately for primary cases, non-primary cases, and unclassified laboratory-confirmed MERS-CoV cases.

## 2. Materials and Methods

### 2.1. Study Design and Participants

Retrospective analysis was performed on epidemiological data of all laboratory-confirmed MERS-CoV cases reported to WHO between 1 January 2015 and 13 April 2018. WHO case definitions for laboratory confirmation for MERS have been previously described [42]. Due to inconsistencies in the collection and reporting of exposure information prior to 2015, our analysis considered cases reported from 1 January 2015. Primary cases were defined as cases with laboratory confirmation of MERS-CoV infection with no direct epidemiological link to a (confirmed or probable) human MERS case. Non-primary cases were defined as cases with laboratory confirmation of MERS-CoV infection, and with a direct epidemiological link to a (confirmed or probable) human MERS case. Unclassified cases are cases with insufficient information reported to WHO, on potential exposures prior to infection, to be classified as a primary or non-primary case.

### 2.2. Ethical Considerations

The case-patient data reported to WHO by member states is anonymized, thus, neither informed consent, nor approval from an institutional review board were required.

### 2.3. Contact with Dromedary Camels as Reported by Member States

Case data reported to WHO includes information on exposures during the 14 days before MERS symptom onset or when laboratory confirmation was reported (i.e., travel history, contact with confirmed or probable human MERS case, any contact with dromedaries, recent healthcare facility visits). Direct exposure to dromedaries was defined as physical contact (e.g., touching, feeding, cleaning, slaughtering, milking, assisting with birth of camelids, or other activities involving physical contact with dromedaries) in the 14 days before symptom onset or when laboratory confirmation was reported. Indirect exposure to dromedaries is defined as visiting camel areas (e.g., markets, racing tracks, farms) without directly touching a camel, or as consumption of dromedary products (e.g., raw/unpasteurized dromedary milk, raw or undercooked dromedary meat, or other products derived from dromedaries, including urine) in the 14 days before symptom onset, or when laboratory confirmation was reported. Any contact with dromedaries is defined as any direct or indirect contact. Direct and indirect contacts were not considered mutually exclusive for the purposes of this analysis as MERS cases may have reported both or either direct and indirect contact with dromedaries prior to symptom onset or laboratory confirmation of infection.

### 2.4. Descriptive Analysis

Descriptive analysis was performed for all MERS-CoV cases reported to WHO between 1 January 2015 and 13 April 2018, using the “epiDisplay” Epidemiological Data Display Package in R, version 3.2.2.0 (https://CRAN.R-project.org/package=epiDisplay).

Descriptive characteristics were expressed as means (SD) and frequencies (%) among all cases, and then separately for primary, non-primary, and unclassified cases. Characteristics and exposures were compared for primary and non-primary cases using Student’s *t* tests for continuous variables, and χ_2_ tests for categorical variables. For all statistical analyses, *p* < 0.05 was considered statistically significant.

## 3. Results

### 3.1. Subject Characteristics

Our analysis considered all 1125 laboratory-confirmed MERS-CoV infections reported to WHO between 1 January 2015 and 13 April 2018. Of the 1125 cases, the mean age was 54.9 (SD 17.3) years, 71.8% were male and 90.0% reported MERS-CoV exposure in KSA. Of the 348 primary cases, the mean age was 57.7 (SD 15.8) years, 86.5% were male and 96.3% reported MERS-CoV exposure in KSA (Table 1). Table 1 provides further description of laboratory-confirmed MERS-CoV infections reported to WHO between 1 January 2015 and 13 April 2018.

Among the included cases, 348 (30.9%) were classified as primary cases, 455 (40.4%) were non-primary cases, and 322 (28.6%) were unable to be classified. In total, there were 414 (36.8%) deaths, of which 151 (36.5%) were among primary cases, 149 (36.0%) among non-primary cases, and 114 (27.5%) among unclassified cases.

### 3.2. Direct and Indirect Contact with Dromedaries

Table 2 shows contact with dromedaries reported to WHO among all laboratory-confirmed cases, and separately for primary cases, non-primary cases, and unclassified cases between 1 January 2015 and 13 April 2018.

Among primary cases, 191 (54.9%) cases reported any contact with dromedaries: 164 (47.1%) reported direct contact; 155 (44.5%) reported indirect contact. Five (1.1%) non-primary cases reported contact with dromedaries: both direct and indirect contact. For all, primary, non-primary, and unclassified cases reporting indirect contact with dromedaries, unpasteurized dromedary camel milk was the most frequent type of dromedary camel product consumed.

### 3.3. Comparison of Primary and Non-Primary Cases

Table 3 compares characteristics and exposures of primary and non-primary cases reported to WHO between 1 January 2015 and 13 April 2018. Primary cases were more likely to be older (*p* < 0.001), and with a higher proportion of males compared to non-primary cases (*p* < 0.001). Primary cases were also shown to have greater contact (both direct and indirect) with dromedaries compared to non-primary cases (*p* < 0.001).

## 4. Discussion

This is the first study to describe contact among all MERS-CoV infections reported to WHO with the known animal reservoir of MERS-CoV: dromedary camels. We report that among all of the 1125 MERS-CoV cases reported to WHO between 1 January 2015 and 13 April 2018, 30.9% were primary cases. Among primary cases, 191 (54.9%) reported direct or indirect contact with dromedaries: 164 (47.1%) reported direct, physical, contact with dromedaries, and 155 (44.5%) reported contact with products derived from dromedaries, namely unpasteurized camel milk.

We found primary human cases more likely to be older, with a higher proportion of males compared to all cases, and compared to non-primary or unclassified cases. This likely reflects differences in cultural practices and exposures to dromedaries between men and women in the Middle East, rather than a difference in infection susceptibility. In this study, all primary MERS-CoV infections have occurred in countries in the Middle East, including KSA, which accounts for 96.3% of primary infections reported between 1 January 2015 and 13 April 2018 (Table 1). In this region, dromedary ownership, herding, and farming practices have increased in recent decades, and camel farms are increasingly concentrated close to major cities, with camel workers often living inside or in close proximity to camel barns. As culturally important animals, dromedaries are celebrated in camel races, sales, beauty competitions, and parades, and often kissed, hugged, and greeted, intensifying frequency of direct contact with dromedaries [23,24,39,43]. In addition, unpasteurized camel milk and meat are widely consumed, despite current WHO recommendations for people living in areas with reported MERS-CoV circulation to avoid drinking raw camel milk [44], and camel urine, which is believed to have therapeutic benefits. The risk of MERS-CoV infection from the consumption of unpasteurized camel milk has been evaluated in Qatar, and the authors found evidence of MERS-CoV RNA and neutralizing antibodies in the milk, but could not determine if MERS-CoV was in the milk or contaminated during the milking process [35].

Although it is clear that contact with infected dromedaries are the primary source of recurrent introduction of MERS-CoV into the human population, mitigating spillover from dromedaries to humans has been limited by a lack of clarity on the modes of transmission between dromedaries and humans, the extent of spillover to humans, and the epidemiology of MERS-CoV circulation in dromedaries in large parts of Africa and South Asia. A deeper understanding of why zoonotic transmission has been undetected in many countries in Africa, the Middle East (outside the Arabian Peninsula), and South Asia, despite high seroprevalence in dromedaries in such countries, is required [45]. WHO, the Food and Agriculture Organization of the United Nations (FAO), in collaboration with technical partners in these regions, are currently working to implement field studies at the animal/human interface, to further understand the extent of circulation in dromedaries, zoonotic transmission, dromedary husbandry practices, and trade patterns of dromedaries in a number of countries across Africa and South Asia (personal communication, with permission, Van Kerkhove).

Our study applied a One Health vision to retrospective analysis of epidemiological data to determine if we could better understand infection at the animal/human interface. The findings show clearly that contact with dromedaries has likely played an important role in the continued introduction of MERS-CoV into the human population from the dromedary camel reservoir. While there have been notable improvements in surveillance and reporting of human cases since 2015, multidisciplinary research, cross-sectoral collaboration at country level, public awareness about the disease, and laboratory and surveillance capacity in affected countries, particularly since 2015, there is still a need to further understand frequency and patterns of contact between infected dromedaries and humans that lead to zoonotic transmission, best achieved through multisite anthropological studies in areas across which MERS-CoV is known to circulate, not only in human populations, but also in dromedary populations. Interrupting zoonotic transmission could also be achieved through the ongoing development and application of dromedary and/or human vaccine candidates.

The results of our study are strengthened by the size of the study, which includes all laboratory confirmed cases reported to WHO since 1 January 2015. We were not able include all laboratory confirmed cases reported to WHO since 2012, because prior to 2015, there were inconsistencies in the way exposure information for each human MERS-CoV infection was collected. For example, at the start of this epidemic in 2012, a comprehensive data collection tool was not used by all countries identifying MERS cases and potential risk factor data, and disease/outcome information about individual patients after the time of reporting was not systematically reported to WHO. Even among data reported since 2015, there is some missing data for contact with dromedaries and there is a complete absence of information on the use of personal protective equipment (PPE; e.g., gloves, boots, coveralls, masks/respirators) when in direct contact with dromedaries, and on hygiene practices following contact with dromedaries. This limits our ability to draw conclusions from our dataset, as to how each case was infected and the exact route(s) of transmission. The use of PPE, however, has been evaluated in a detailed case-control study in Qatar evaluating specific types of dromedary contact among seropositive vs seronegative occupational workers, which found that hand washing before and after contact with the dromedary was protective against infection with MERS-CoV [46].

Our dataset is also limited by our ability in classifying cases based on available information reported to WHO at the time of reporting by the country. For example, thorough outbreak investigations, which include full genome sequencing of the virus, may find that cases which were initially classified as non-primary cases, may in fact be primary cases, and this information was not regularly relayed to WHO. More complete case reporting, including exposures prior to symptom onset, would improve our ability to assess non-human exposures that may have led to primary MERS illness in humans. Efforts are currently being made to retrospectively review and update the epidemiological data for all cases reported to WHO to date, particularly prior to 2015. To aid Member States in more systematic data collection on suspected and confirmed MERS cases, WHO has updated guidance on investigation of cases, and has revised the MERS case reporting forms, which include specific questions about contact with known MERS patients, healthcare visits, travel, occupation, dromedary contact, other animal contact, and underlying medical conditions within the 14 days prior to symptom onset [47,48].

## 5. Conclusions

In conclusion, a lack of systematic reporting on exposures and risk factors, including contact with dromedaries for each MERS case identified since 2012, prevents a clear understanding of how infection occurred in each case. However, it is clear from the data reported that contact with dromedaries has played an important role in transmission of MERS-CoV into the human population from the dromedary reservoir. As a result, further understanding the geographic scope of MERS-CoV circulation in dromedaries, and limiting direct and indirect contact with infected dromedaries, remains important for reducing zoonotic transmission of MERS-CoV.

## Figures and Tables

**Table 1:** Description of laboratory-confirmed MERS-CoV cases reported to World Health Organization (WHO) between 1 January 2015 and 13 April 2018 (*n* = 1125). Results are presented as frequencies (%), unless otherwise stated.

Characteristics		All Cases (*n* = 1125)		Primary Cases (*n* = 348)		Non-Primary Cases (*n* = 455)		Unclassified Cases (*n* = 322)	
		*n*	%	*n*	%	*n*	%	*n*	%
**Age**									
	Mean (years)	54.9		57.7		53.4		54.1	
	SD (years)	17.3		15.8		17.9		17.6	
**Sex**									
	Male	808	71.8	301	86.5	284	62.4	223	69.3
	Female	313	27.8	45	12.9	171	37.6	97	30.1
	Not reported	4	0.4	2	0.6	0	0	2	0.6
**Country of MERS-CoV exposure**									
*Within the Middle East*									
	Islamic Republic of Iran	1	0.1	0	0	1	0.2	0	0
	Jordan	13	1.2	0	0	4	0.9	9	2.8
	Kingdom of Saudi Arabia	911	90.0	335	96.3	276	60.7	300	93.2
	Kuwait	2	0.2	2	0.6	0	0	0	0
	Oman	10	0.9	4	1.2	1	0.2	5	1.6
	Qatar	9	0.8	5	1.4	0	0	4	1.2
	United Arab Emirates	6	0.5	2	0.6	0	0	4	1.2
*Outside the Middle East*									
	Republic of Korea	172	15.3	0	0	172	37.8	0	0
	Not reported	1	0.1	0	0	1	0.2	0	0
**Severity**									
	Asymptomatic/mild	99	8.8	7	2.0	64	14.1	28	8.7
	Pneumonia/non-ICU	241	21.4	102	29.3	54	11.9	85	26.4
	Severe/fatal	553	49.2	209	60.1	190	41.8	154	47.8
	Not reported	232	20.6	30	8.6	147	32.3	55	17.1
**Outcome**									
	Survived	245	21.8	79	22.7	109	24.0	57	17.7
	Died	414	36.8	151	43.4	149	32.8	114	35.4
	Not reported	466	41.4	118	33.9	197	43.3	151	46.9

MERS-CoV: Middle East respiratory syndrome coronavirus; non-ICU: non-intensive care unit; primary case = cases with laboratory confirmation of MERS-CoV infection with no direct epidemiological link to a (confirmed or probable) human MERS case; non-primary case = cases with laboratory confirmation of MERS-CoV infection and with a direct epidemiological link to a (confirmed or probable) human MERS case; unspecified case = cases with insufficient information reported to WHO, on potential exposures prior to infection, to be classified as a primary or non-primary case.

**Table 2:** Direct and indirect contact with dromedaries among laboratory-confirmed MERS-CoV cases reported to WHO between 1 January 2015 and 13 April 2018, *n* = 1125. Results are presented as frequencies (%), unless otherwise stated.

Contact		All Cases (*n* = 1125)		Primary Cases (*n* = 348)		Non-Primary Cases (*n* = 455)		Unclassified Cases (*n* = 322)	
		*n*	%	*n*	%	*n*	%	*n*	%
**Any contact with dromedaries**									
	Yes	235	20.9	191	54.9	5	1.1	39	12.1
	No	276	24.5	62	17.8	162	35.6	52	16.2
	Not reported	614	54.6	95	27.3	288	63.3	231	71.7
Direct contact with dromedaries *									
	Yes	201	17.9	164	47.1	3	0.7	34	10.6
	No	285	25.3	68	19.5	162	35.6	55	17.1
	Not reported	639	56.8	116	33.3	290	63.7	233	72.4
Indirect contact with dromedaries *									
	Yes	186	16.5	155	44.5	3	0.7	28	8.7
	No	280	24.9	73	21.0	152	33.4	55	17.1
	Not reported	659	58.6	120	34.5	300	65.9	239	74.2
**Type of dromedary product consumed**									
	Unpasteurized milk	163	14.5	137	39.4	3	0.7	23	7.1
	Milk (unspecified)	3	0.3	3	0.9	0	0	0	0
	Meat	2	0.2	2	0.6	0	0	0	0
	Unspecified	18	1.6	13	3.7	0	0	5	1.6

MERS-CoV: Middle East respiratory syndrome coronavirus. * Direct and indirect contact not mutually exclusive—cases may have had both direct and indirect contact with dromedaries.

**Table 3:** Comparison of laboratory-confirmed primary (*n* = 348) and non-primary (*n* = 455) MERS-CoV cases reported to WHO between 1 January 2015 and 13 April 2018. Results are presented as frequencies (%), unless otherwise stated

Characteristics		Primary Cases (*n* = 348)		Non-Primary Cases (*n* = 455)		
		*n*	%	*n*	%	*p* Value
**Age**						
	Mean (years)	57.7		53.4		<0.001
**Sex**						<0.001
	Male	301	86.5	284	62.4	
	Female	45	12.9	171	37.6	
**Outcome**						0.08
	Survived	79	22.7	109	24.0	
	Died	151	43.4	149	32.8	
**Exposures**						
Any contact with dromedaries						<0.001
	Yes	191	54.9	5	1.1	
	No	62	17.8	162	35.6	
Direct contact with dromedaries *						<0.001
	Yes	164	47.1	3	0.7	
	No	68	19.5	162	35.6	
Indirect contact with dromedaries *						<0.001
	Yes	155	44.5	3	0.7	
	No	73	21.0	152	33.4	

MERS-CoV: Middle East respiratory syndrome coronavirus. Primary case = cases with laboratory confirmation of MERS-CoV infection with no direct epidemiological link to a (confirmed or probable) human MERS case; non-primary case = cases with laboratory confirmation of MERS-CoV infection and with a direct epidemiological link to a (confirmed or probable) human MERS case; * Direct and indirect contact not mutually exclusive—cases may have had both direct and indirect contact with dromedaries.
